# Targeting Activated Synovial Fibroblasts in Rheumatoid Arthritis by Peficitinib

**DOI:** 10.3389/fimmu.2019.00541

**Published:** 2019-03-26

**Authors:** Magnus Diller, Rebecca Hasseli, Marie-Lisa Hülser, Iris Aykara, Klaus Frommer, Stefan Rehart, Ulf Müller-Ladner, Elena Neumann

**Affiliations:** ^1^Department of Rheumatology and Clinical Immunology, Campus Kerckhoff, Justus-Liebig-University Giessen, Giessen, Germany; ^2^Department of Orthopedics and Trauma Surgery, Agaplesion Markus Hospital, Frankfurt, Germany

**Keywords:** JAK inhibition, peficitinib, synovial fibroblast, rheumatoid arthritis, IL-1β

## Abstract

**Background:** Synovial fibroblasts (SF) play a major role in the pathogenesis of rheumatoid arthritis (RA) and develop an aggressive phenotype destroying cartilage and bone, thus termed RASF. JAK inhibitors have shown to be an efficient therapeutic option in RA treatment, but less is known about the effect of JAK inhibitors on activated RASF. The aim of the study was to examine the effects of JAK inhibitors on activated RASF.

**Methods:** Synovium of RA patients was obtained during knee replacement surgeries. Synoviocytes were isolated and pretreated with JAK inhibitors. Pro-inflammatory cytokines and matrix degrading proteinases were measured by ELISA in supernatant after stimulation with oncostatin M or IL-1β. The proliferation of RASF was measured by BrdU incorporation. Cell culture inserts were used to evaluate cell migration. For adhesion assays, RASF were seeded in culture plates. Then, plates were extensively shaken and adherent RASF quantified. Cell viability, cytotoxicity and apoptosis were measured using the ApoTox-Glo™ Triplex and the CellTox™ Green Cytotoxicity Assay.

**Results:** Tofacitinib and baricitinib decreased the IL-6 release of RASF stimulated with oncostatin M. JAK inhibition attenuated the IL-6 release of IL-1β activated and with soluble IL-6 receptor treated RASF. In contrast, only peficitinib and filgotinib decreased the IL-6 release of RASF activated with IL-1β. Peficitinib decreased also the MMP-3, CXCL8, and CXCL1 release at 5 μM. Moreover, peficitinib was the only JAK inhibitor suppressing proliferation of activated RASF at 1 μM. Peficitinib further decreased the migration of RASF without being cytotoxic or pro-apoptotic and without altering cell adhesion.

**Conclusions:** JAK inhibitors effectively suppress the inflammatory response induced by oncostatin M and by transsignaling of IL-6 in RASF. Only peficitinib modulated the IL-1β-induced response of RASF and their proliferation *in vitro* at concentrations close to reported Cmax values of well tolerated doses *in vivo*. In contrast to filgotinib, peficitinib also highly suppressed RASF migration showing the potential of peficitinib to target RASF.

## Introduction

Rheumatoid arthritis (RA) is the most frequent disease among the inflammatory rheumatic diseases and affects approximately 0.41 to 0.54% of the adult population in the US (1). Currently, the pharmacological treatment options include conventional synthetic (cs), biological (b), and targeted synthetic (ts) DMARDs (disease-modifying antirheumatic drugs) (2). In 2012, tofacitinib was the first FDA approved Janus kinase inhibitor (JAKi) and the first tsDMARD for treatment of rheumatoid arthritis (RA) in the US. The approval of baricitinib followed in 2018. In the EU, baricitinib and tofacitinib were both approved for treatment of RA in 2017. In the past years, additional JAKi were developed. They differ mainly in the inhibition profile of the four isoforms JAK1, JAK2, JAK3, and TYK2. Filgotinib, inhibiting mainly JAK1/2 and the panJAKi peficitinib are currently examined in clinical trials. Both of the latter inhibitors are well tolerated and high C_max_ (maximum serum concentration) values above 1 μM could be reached in clinical studies (3, 4).

The effect of JAKi is mediated by suppression of the intracellular signaling of cytokines depending on JAK phosphorylation. IL-6, granulocyte-macrophage colony-stimulating factor (GM-CSF), and interferons (IFNs) are the most important representatives of these cytokines being involved in the pathogenesis of RA (5). Specifically activated lymphocytes are targeted by tofacitinib showing decreased proliferation and a suppressed production of IL-17 and IFNγ after treatment (6).

Synovial fibroblasts (RASF) play a major role in RA by contributing to the growing pannus in the inflammatory milieu of joints (7). Furthermore, they develop an aggressive phenotype, and are able to migrate to and invade into healthy cartilage (7, 8). Tofacitinib has shown to selectively block activated pathways dependent on the phosphorylation of JAKs, e.g., oncostatin M in RASF (9). However, RASF are mainly activated by the inflammatory milieu with IL-1β and TNF-α being central cytokines. Of note, JAKs are primarily not required for mediating the effects of IL-1β on gene expression of e.g., pro-inflammatory cytokines (10, 11). Nevertheless, TNF-α induces the production of pro-inflammatory cytokines elevating the pro-inflammatory response of RASF in an autocrine manner. These secondary effects of e.g., IFN could be suppressed by JAKi and therefore reduce the general pro-inflammatory response of TNF-α treated RASF (12). In contrast, tofacitinib did not affect the IL-6 and IL-8 release of IL-1β activated RASF (6). The effect of other JAKi has not yet been examined. Taken together, little is known about the impact especially of new developed JAKi (peficitinib, filgotinib) on the pro-inflammatory response of IL-1β activated RASF and on their proliferation. Therefore, the aim of the study was to compare the effect of different JAKi on pro-inflammatory response of activated RASF and further characterize the effect of the most effective inhibitors.

## Materials and Methods

### Cells

Synovium of patients suffering from RA was obtained during knee replacement surgery (Agaplesion-Markus-Hospital, Frankfurt). All patients fulfilled the classification criteria of the American College of Rheumatology (13). This study was carried out in accordance with the recommendations of the ethic committee of the University of Giessen. All subjects gave written informed consent in accordance with the Declaration of Helsinki. The protocol was approved by the ethic committee of the University of Giessen. Synovium samples were digested (1 h at room temperature, dispase-II-solution, 0.1 ml/ml, PAN-Biotech, Aidenbach, Germany) (14) and passed through cell strainers. After centrifugation, cells were cultured in DMEM (GE Healthcare, Germany) containing 10% heat-inactivated fetal calf serum (FCS, Sigma-Aldrich, Taufkirchen, Germany), 1 U/ml penicillin/streptomycin (AppliChem, Darmstadt, Germany), and 1 mM HEPES (GE Healthcare) at 37°C and 10% CO_2_ (14). Cells were passaged using trypsin/ EDTA (Capricorn, Ebsdorfergrund, Germany) for a maximum of passage 7 (15). Human umbilical vein endothelial cells (HUVEC) were cultured in Endothelial Cell Growth Medium 2 with recommended supplement mix (both PromoCell, Heidelberg, Germany) containing 1 U/ml penicillin/streptomycin and 1 mM HEPES at 37°C and 5% CO_2_.

### Stimulation of RASF

RASF were seeded into 6-well plates (1 × 10^5^ cells/well) and pretreated with JAKi (Selleckchem, Houston, USA) for 2 h and then additionally stimulated with IL-1β (R&D systems, Wiesbaden, Germany) or oncostatin M (OSM, R&D systems) for the indicated time. Experiments with RASF stimulated with IL-1β and sIL-6R (R&D systems) were performed in 24-well plates (2 × 10^4^ cells/well). Supernatants were collected, centrifuged at 10,600 × g for 10 min and stored at −20°C. Vehicle control containing 0.1% DMSO served as a control. Synthesis of pro-inflammatory cytokines, matrix degrading proteinases (MMP), and chemokines were measured by commercially available enzyme-linked immunosorbent assay (ELISA, R&D systems) according to manufacturer's protocol.

### Migration Assay

To determine the impact of peficitinib on migration of RASF, 1 × 10^5^ cells were seeded in Corning® Transwell® polycarbonate membrane cell culture inserts for 24-well plates (Corning, New York, USA) containing a final concentration of 2% FCS. The lower chamber contained 10% FCS as chemoattractant. The non-migrated cells on top of the membrane were removed by gently wiping the membrane with a cotton swab. The migrated cells on the bottom side were stained with 4′,6-diamidino-2-phenylindole (DAPI) and the number of RASF was counted in 5 representative areas of 3 wells (200x magnification).

### Adhesion Assays

RASF were pretreated with peficitinib, filgotinib or DMSO 0.1% and additionally stimulated with IL-1β as described previously. The cells were detached with accutase (Capricorn), centrifuged and seeded in 24-well plates at 1 × 10^4^ cells/well. After incubation for 1 h at 37°C, the plate was shaken for 5 min at full speed and the non-adherent or weakly attached cells were removed by washing with PBS. The procedure was repeated twice. The remaining attached RASF were stained with 0.1% crystal-violet dye in methanol for 10 min and counted in 4 representative areas of 3 wells per cell population (50x magnification).

For the cell-to-cell binding assay HUVECs were seeded in 48-well plates and cultured as described above until the confluence reached 100%. RASF were pretreated with JAKi as described previously. In the last 30 min Calcein-AM (Thermo Fisher Scientific, Waltham, USA) was supplemented to the medium according to the manufacturer's instructions. Cells were detached with accutase, counted and 5 × 10^3^ cells were seeded on top of the endothelial cell layer. After 30 min, plates were shaken for 5 min at full speed and the non-adherent or weakly attached cells were removed by washing with PBS. The procedure was repeated twice. Remaining cells were fixed with 4% formaldehyde and green fluorescent (Calcein-AM stained) RASF were counted in 4 representative areas of 3 wells per cell population (50x magnification).

### Proliferation Assay

RASF were seeded in 96-well plates at 3.5 × 103 cells/well overnight and then treated with JAKi or DMSO 0.1% with/without IL-1β for 24 h in the presence of 5-bromo-2′-deoxyuridine (BrdU). The BrdU incorporation indicating the proliferation of RASF was determined by a commercially available calorimetric BrdU cell proliferation assay kit (Merck, Darmstadt, Germany) according to manufacturer's protocol.

### Measurement of Apoptosis, Viability, and Cytotoxicity

For measurement of changes in levels of apoptosis, viability, and cytotoxicity caused by peficitinib, RASF were seeded in 96-well plates at 3.5 × 103 cells/well overnight and then treated with peficitinib for 19 and 38 h. The ApoTox-Glo™ Triplex Assay (Promega, Madison, USA) was performed according to manufacturer's protocol. For the detection of cytotoxicity at multiple time points the CellTox™ Green Cytotoxicity Assay was used. The fluorescence (viability, cytotoxicity) and the luminescence (apoptosis) were measured as indicated by the manufacturer.

### Statistics

All data are presented as arithmetic mean ± standard deviation (SD). For comparisons with a single control group, a one way ANOVA followed by Dunnett's *post-hoc* test was performed. The assessment of significance level for pair wise comparisons was calculated by a Student two-tailed *t*-test and Mann-Whitney-*U*-Test. *P* < 0.05 were considered significant. Statistical calculations and graphics were performed using GraphPad Prism.

## Results

### Tofacitinib and Baricitinib Attenuated IL-6 and OSM Dependent IL-6 Release

**Figure 1:**
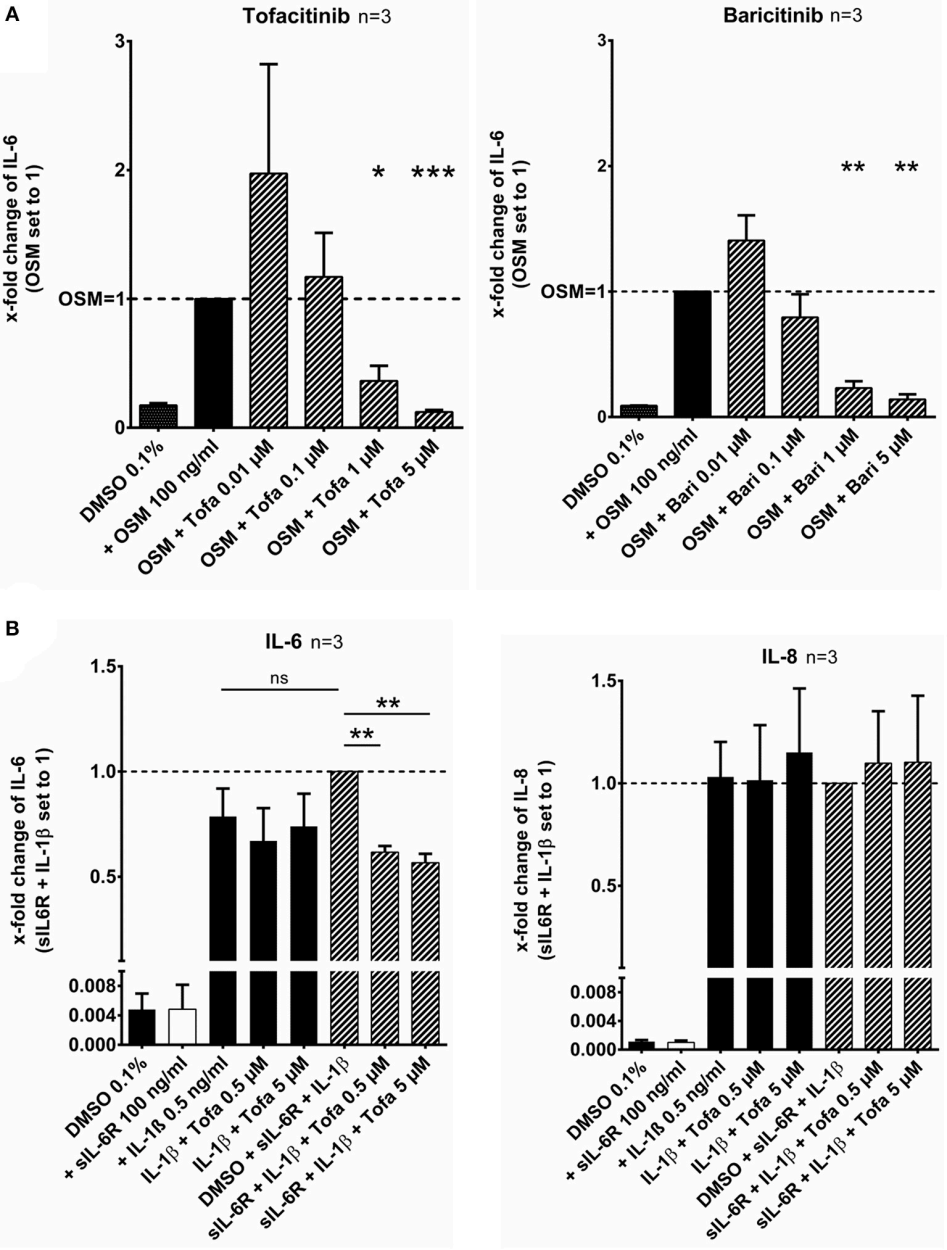
Effect of tofacitinib and baricitinib on IL-6 dependent release of cytokines. **(A)** RASF were pretreated with JAKi or vehicle control (DMSO 0.1%) for 2 h and then additionally activated with OSM (100 ng/ml) for 24 h. The IL-6 release was decreased by tofacitinib or baricitinib confirming the inhibition of JAK dependent pathway. **(B)** The addition of sIL-6R to IL-1β stimulated RASF leads to an increase of IL-6 but not of IL-8 release. This increase could be blocked by tofacitinib. ^*^p < 0.05, ^**^p < 0.01, and ^***^p < 0.001 compared with OSM **(A)** or IL-1β and sIL6R **(B)** stimulated cells.

RASF were pretreated with tofacitinib or baricitinib for 2 h and then additionally stimulated with the IL-6 like cytokine oncostatin M (OSM, 100 ng/ml) for 24 h (Figure 1A). Tofacitinib decreased the induced IL-6 release by 64% at 1 μM and by 88% at 5 μM compared to OSM alone (*p* < 0.05, *p* < 0.001, *n* = 3, Figure 1A). Baricitinib at 1 and 5 μM also attenuated the IL-6 release by 77 and 86% (both *p* > 0.05, *n* = 3, Figure 1A). The combination of the soluble IL-6 receptor (sIL-6R) and IL-1β increased the IL-6 release by 21% (not significant) but not the IL-8 release in comparison to the stimulation with IL-1β alone (Figure 1B). The effect caused by sIL-6R was completely blocked by 0.5 or 5 μM tofacitinib (both *p* < 0.01).

### Effect of JAKi on Cytokine and MMP Release of RASF Activated by IL-1β

**Figure 2:**
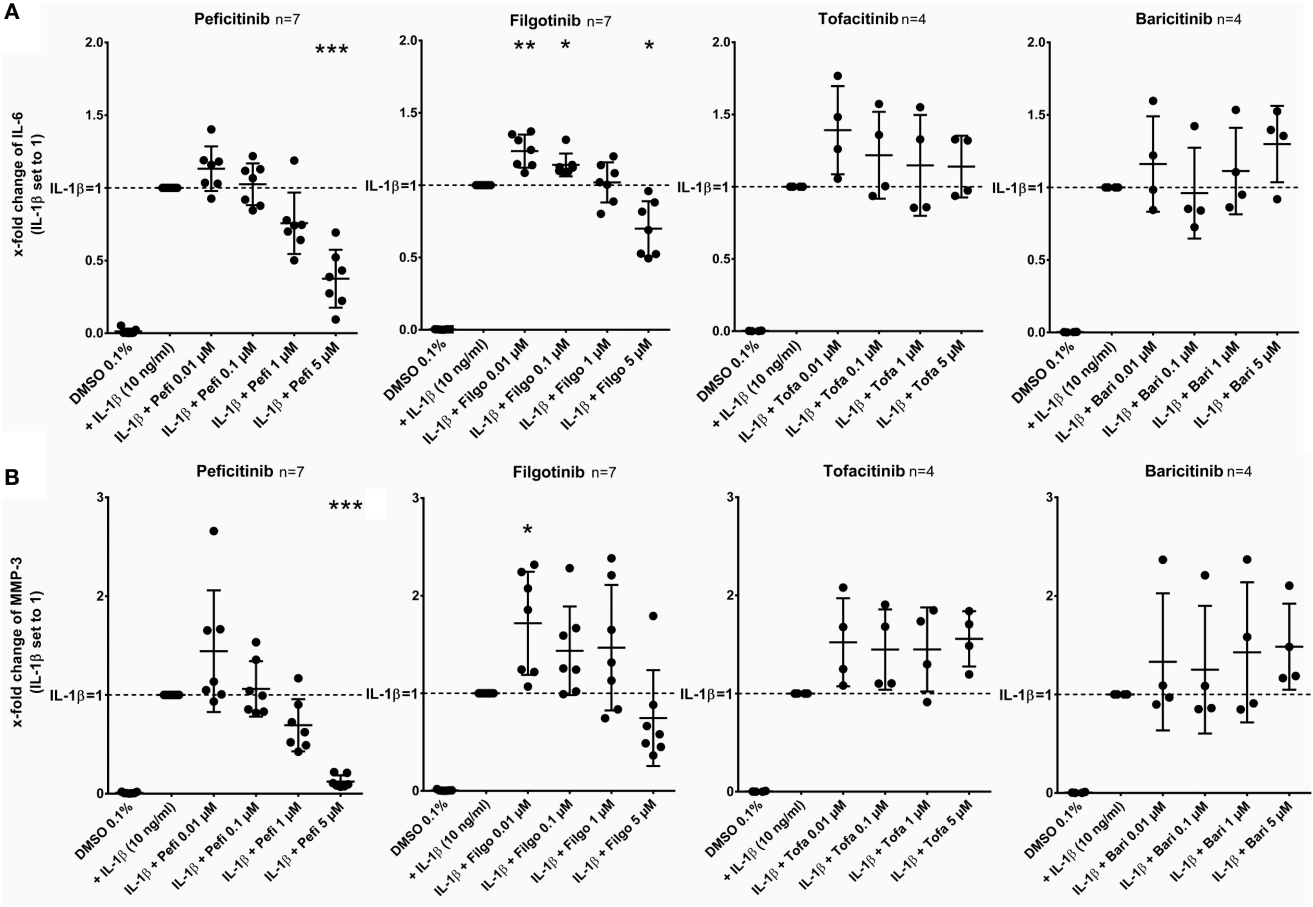
Effect of JAKi on IL-6 and MMP-3 release of activated RASF by IL-1β. RASF were pretreated with JAKi or vehicle control (DMSO 0.1%) for 2 h and then additionally activated with IL-1β (10 ng/ml) for 17 h. The IL-6 and MMP-3 release was decreased by peficitinib at 1 and 5 μM, whereas filgotinib only decreased IL-6 levels at 5 μM. Tofacitinib and baricitinib did not attenuate the release of both proteins. ^*^p < 0.05, ^**^p < 0.01, and ^***^p < 0.001 compared with stimulated cells by IL-1β.

After stimulation of RASF with IL-1β (10 ng/ml) for 17 h, peficitinib and filgotinib decreased the IL-6 release by 62% (*p* < 0.001) and by 30% at 5 μM (*p* < 0.05, *n* = 7). Peficitinib also attenuated the IL-6 release at 1 μM (24%, *n* = 7), but this observation did not reach the significance level due to high variability in responsiveness of RASF from different patients. Filgotinib even slightly elevated IL-6 levels at 0.01 μM (24%, *p* < 0.01) and at 0.1 μM (14%, *p* < 0.05) (Figure 2A).

Peficitinib at 5 μM reduced the MMP-3 levels induced by IL-1β by 88% (*n* = 7, *p* < 0.001). At 1 μM, 6 of 7 patients showed a decrease (reduction by 31%, *n* = 7, not significant). The variability of MMP-3 levels was high after treatment with filgotinib and we could not observe a significant reduction (Figure 2B).

In contrast, tofacitinib and baricitinib did not decrease the IL-6 or MMP-3 release.

### Effect of JAKi on Chemokine Release of RASF Activated by IL-1β

**Figure 3:**
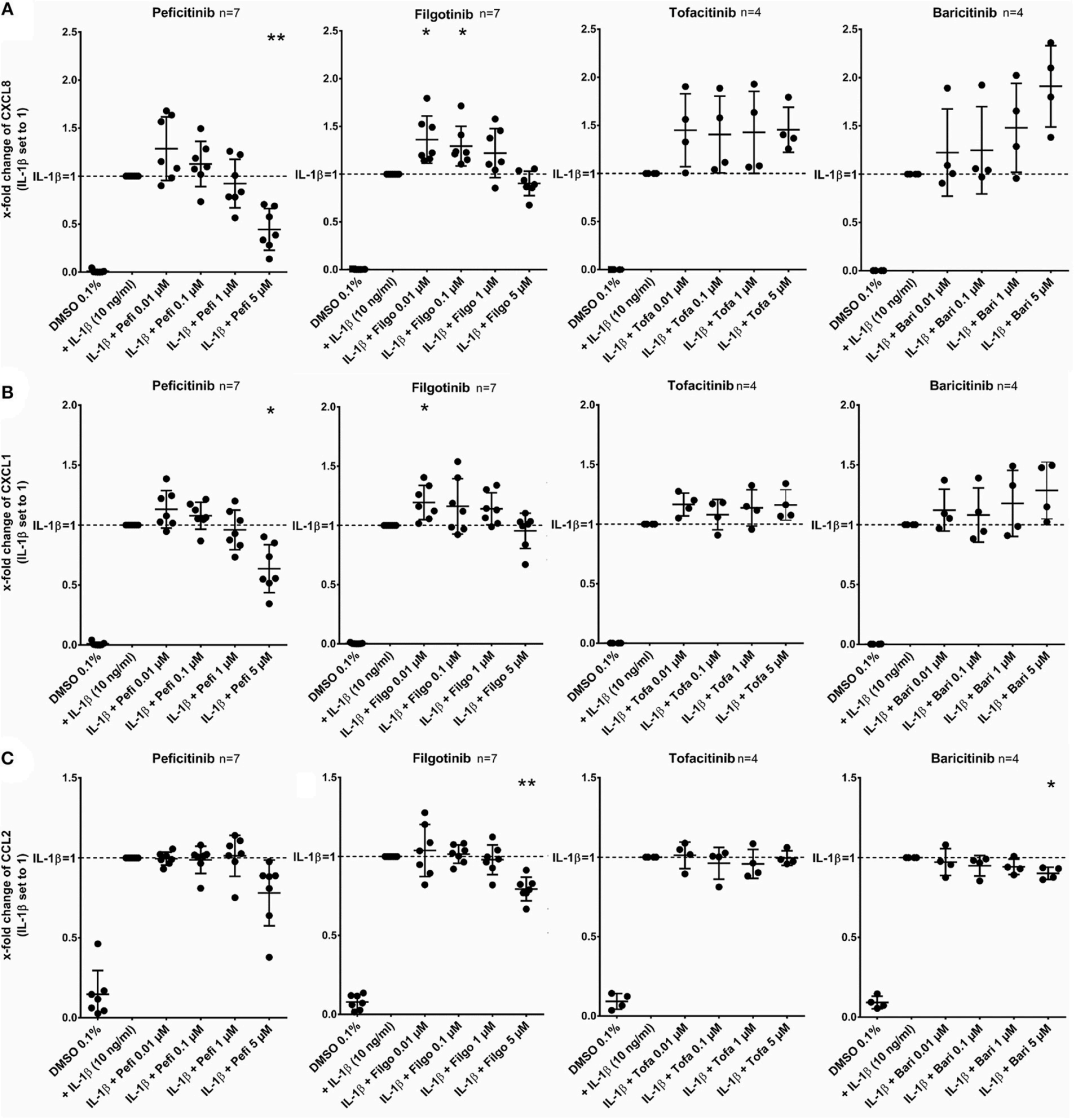
Effect of JAKi on chemokine release of activated RASF by IL-1β. RASF were pretreated with JAKi or vehicle control (DMSO 0.1%) for 2 h and then additionally activated with IL-1β (10 ng/ml) for 17 h. Peficitinib at 5 μM decreased the release of CXCL8 **(A)** and CXCL1 **(B)**. CCL2 was attenuated significantly by filgotinib and baricitinib **(C)**. ^*^p < 0.05 and ^**^p < 0.01 compared with stimulated cells by IL-1β.

In contrast to other JAKi, only peficitinib at 5 μM decreased the release of CXCL8 (56%, *p* < 0.01) and CXCL1 (36%, *p* < 0.05). CCL2 was attenuated by peficitinib (22%) and by filgotinib (21%), whereas only the decrease of filgotinib was significant (*p* < 0.01). Baricitinib also attenuated the CCL2 release at 5 μM but compared to the other tested JAKi the effect was low (10%, *p* < 0.05) (Figure 3).

### Peficitinib and Filgotinib Reduced Migration of RASF

**Figure 4:**
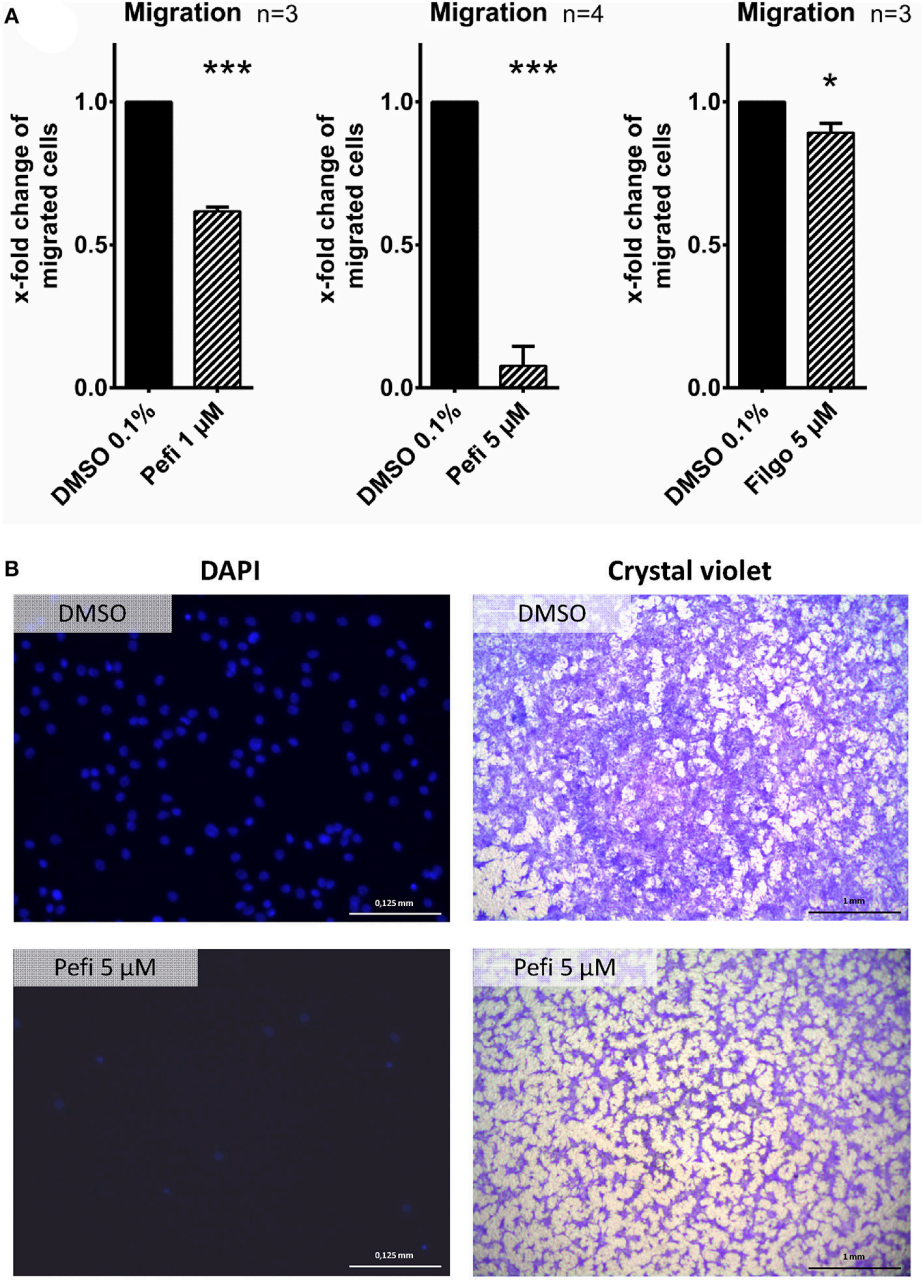
Peficitinib and filgotinib attenuated migration of RASF. **(A)** The migration of RASF through a membrane (8 μM pores) toward a FCS gradient was reduced by pefictinib at 1 and 5 μM. **(B)** Representative crystal violet and DAPI staining of the bottom side of the inserts are shown. DAPI staining was used to quantify the migrated cells. ^*^p < 0.05 and ^***^p < 0.001 compared with vehicle control.

**Figure 5:**
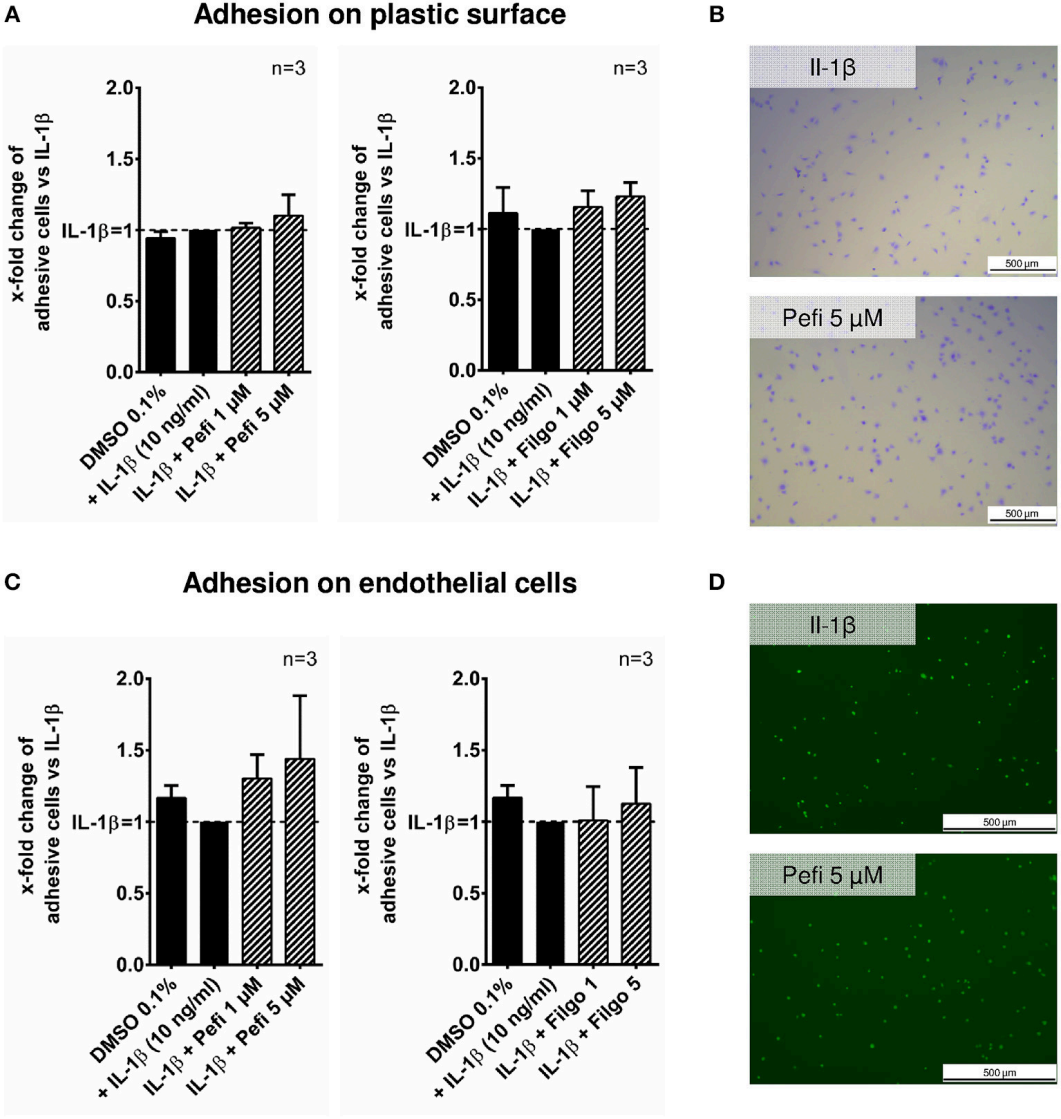
Effect of different JAKi on proliferation of RASF. The adhesion of RASF on plastic surface **(A)** and on endothelial cells (HUVECs) **(C)** was not influenced by IL-1β and peficitinib or filgotinib at 1 and 5 μM. **(B)** Representative crystal violet staining is shown. for adhesive cells on plastic surface. **(D)** Attached Calcein-AM stained RASF on endothelial cells were counted by fluorescence microscopy.

The effect of peficitinib on migration of RASF toward a FCS gradient was studied by inserts containing membranes with 8 μm pores. Peficitinib decreased the number of migrated cells by 38% at 1 μM and by 92% at 5 μM (both *p* < 0.001, Figure 4A). In contrast, filgotinib only attenuated migration by 7% at 5 μM (*p* < 0.05, Figure 4A). DAPI staining was performed to count the migrated cells (Figure 4B). The effect on migration was not due to changed adhesion, because the short-term adhesion toward the plastic surface even of IL-1β activated RASF was not changed significantly by peficitinib or by filgotinib (Figures 5A,B). The adhesion on endothelial cells (HUVECs) was also not significantly affected by both JAKi (Figures 5C,D).

### Effect of Different JAKi on Proliferation of RASF

**Figure 6:**
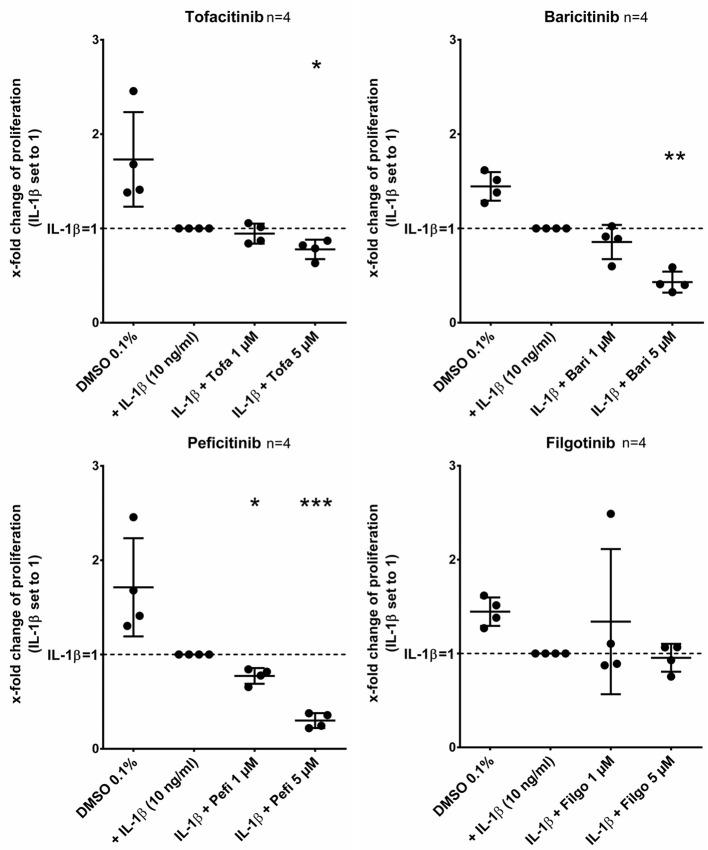
Peficitinib did not influence the adhesion of activated RASF. Tofacitinib and baricitinib at 5 μM decreased proliferation of RASF activated by IL-1β, whereas filgotinib did not affect the proliferation. In contrast to the other JAKi, peficitinib inhibited proliferation of RASF at 1 μM. The proliferation was measured by BrdU-incorporation over 24 h. ^*^p < 0.05, ^**^p < 0.01, and ^***^p < 0.001 compared with activated RASF by IL-1β.

Tofacitinib, baricitinib, and peficitinib at 5 μM decreased the proliferation rate studied by BrdU incorporation after 24 h compared to IL-1β (Figure 6). The strongest effect with a reduction of about 70% (*p* < 0.001, *n* = 4) was observed with peficitinib. Furthermore, only peficitinib attenuated the proliferation at 1 μM (23%, *p* < 0.05, *n* = 4). Interestingly, filgotinib did not change the proliferation of RASF.

### Peficitinib Did Not Act Cytotoxic or Pro-apoptotic on RASF

**Figure 7:**
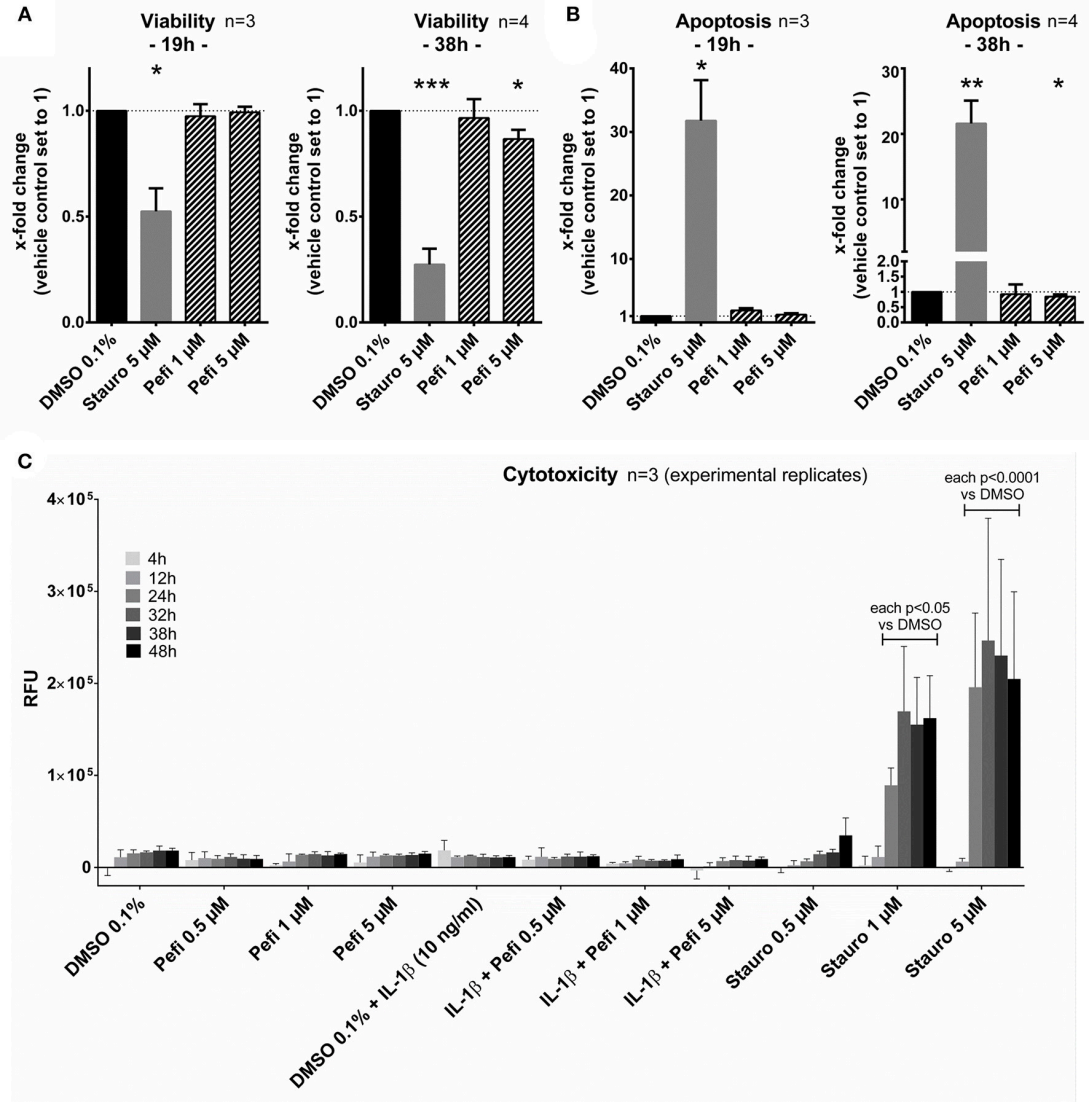
Peficitinib did not act cytotoxic or pro-apoptotic on RASF. **(A)** The viability of RASF was not changed after treatment with peficitinib for 19 h. After 38 h the viability was slightly decreased by 5 μM peficitinib. **(B)** Even after 38 h, peficitinib did not affect the apoptosis rate of RASF measured by luciferase based detection of caspase-3/7 activation. **(C)** Peficitinib at 0.5, 1, and 5 μM alone or in combination with IL-1β did not induce cytotoxicity between 4 and 48 h. For all experiments staurosporin served as positive control. ^*^p < 0.05, ^**^p < 0.01 and ^***^p < 0.001 compared with vehicle control.

The viability, activation of caspase 3/7 and the membrane integrity was measured to exclude toxic or pro-apoptotic effects of peficitinib. The viability did not decrease after 19 h treatment of RASF with peficitinib at 1 and 5 μM in contrast to the staurosporin control (Figure 7A). After 38 h peficitinib at 5 μM induced a slight reduction of 13% (*p* < 0.05, *n* = 4). This effect was not caused by apoptosis after 19 or 38 h (Figure 7B) or cytotoxic effects between 4 and 48 h in contrast to the staurosporin control (Figure 7C).

## Discussion

In this study, all tested JAKi showed the ability to suppress the IL-6 and OSM mediated pro-inflammatory response in RASF. Furthermore, the panJAKi peficitinib was able to attenuate the broad pro-inflammatory response and the proliferation of IL-1β activated RASF.

First, we confirmed that tofacitinib is able to suppress the effects of the IL-6 like cytokine oncostatin M on IL-6 release. In contrast to findings of Migita et al. no reduction was observed in our study using 0.1 μM tofacitinib (9). This can be explained by differences in the experimental settings of both studies: We did not incubate the cells in serum-free medium and used higher concentrations of OSM (100 vs. 20 ng/ml). Additionally, we could show that baricitinib decreased OSM-mediated IL-6 release besides the known downregulation of CCL2 (16). RASF do not express the sIL-6R (17) and only transsignaling appears to play a role of mediating IL-6 effects in RASF (18, 19). Therefore, we additionally treated IL-1β activated RASF with sIL-6R, and indeed we could observe an increase of the IL-6 release. This increase could be fully blocked by treatment with tofacitinib even at 0.5 μM. These findings confirm that JAKi suppress the JAK dependant signaling of the corresponding cytokines in RASF.

The effect of IL-6 and sIL-6R appears to be selective as the IL-1β induced IL-8 release could not be further increased by treatment with sIL-6R and not be blocked by JAKi. This finding is in line with data from Rosengren et al. showing that the TNF-α effect on IL-8 release was also not attenuated by JAK inhibition (12). However, in whole synovial tissue, the IL-8 levels were reduced in RA-patients treated with tofacitinib (20). This indicates that RASF are not the main producers of IL-8 in the synovial tissue or are affected by declining inflammatory mediators of other immune cells targeted by tofacitinib.

RASF cause a release of pro-inflammatory cytokines elevating the inflammatory response in an autocrine manner. This impact of secondary released mediators is known for the effect of TNF-α and IL-1β on IP-10 expression (12). Especially, type I interferons appear to mediate those secondary effects. Correspondingly, tofacitinib decreased the TNF-α induced release of CCL2 and IP-10 by suppression of JAK signaling (12). However, it has been also described that tofacitinib did not attenuate the IL-6 release of RASF stimulated with IL-1β (6). We confirmed this observation and could additionally show that baricitinib as well as tofacitinib did not suppress the IL-1β mediated IL-6, MMP3, CXCL8, and CXCL1 release. These data indicate that the effects of JAK-dependant cytokines like IL-6 or OSM on IL-6, MMP3, CXCL8, and CXCL1 release appear to be negligible in the presence of high concentrations of IL-1β or in absence of sIL-6R. Against the background of the strong heterogenic distribution of sIL-6R levels with mean levels of 76 ng/ml in synovial fluid of RA patients (21), the treatment failure of some patients treated with JAKi could be explained by effects of remaining local high levels of IL-1β affecting RASF. Conversely, the successful treatment could partly be explained by suppressing transsignaling in RASF in the case of high sIL-6R levels and low IL-1β levels.

Of note, peficitinib and filgotinib were able to attenuate the IL-1β effect, but only above concentrations of 1 μM. Both inhibitors decreased the IL-6 release induced by IL-1β at 5 μM. Peficitinib also suppressed the IL-6 release at 1 μM in RASF from 6 to7 patients. Similar results for peficitinib were observed for the MMP-3 and CXCL1 release. In contrast, filgotinib did not affect the MMP-3 or CXCL1 levels but decreased CCL2 levels. Peficitinib was also the only JAKi decreasing the proliferation of IL-1β activated RASF at 1 μM. The effects of baricitinib and tofacitinib occurred even at 5 μM, but the C_max_ levels of both JAKi are below 200 nM after oral intake of approved doses (22, 23). In contrast, peficitinib is well tolerated by oral application of dosages up to 100 mg twice daily which cause serum levels up to 1.14 μM (373 ng/ml) (3). For filgotinib, C_max_ values up to 3.36 μM after application of 200 mg once daily were reached (4).

Additionally, peficitinib decreased impressively the migration of RASF even at 1 μM without affecting the adhesion. Taken together, peficitinib appears to be superior to other JAKi in decreasing the inflammatory response and the proliferation of RASF, although all JAKi effectively reduce the direct JAK-mediated signaling induced by OSM and IL-6/sIL-6. However, the effect of peficitinib occurs mainly at high concentrations and so we excluded possible pro-apoptotic and cytotoxic effects. The slightly decrease of viability observed after 38 h is not mediated by cytotoxicity or apoptosis and could be explained by decreased proliferation.

At concentrations higher than 1 μM, the used JAKi should inhibit JAK1, 2, and 3. For example, tofacitinib, and baricitinib at 1 μM were both able to inhibit sufficiently OSM-induced phosphorylation of JAK1, 2, and 3 (16). At concentrations of 1–5 μM used in our study, differences of JAKi in the ability to block different isoforms are not most likely to explain our observed differences. Filgotinib is described as a JAK1/2 inhibitor with comparable IC50 (inhibitory concentration 50%) values for JAK1/2 but even higher IC50 for the other JAKs in comparison to tofacitinib (24, 25). However, filgotinib slightly decreased the IL-6 release at 5 μM in contrast to tofacitinib. Furthermore, the nearly completely reduction of MMP-3 to baseline by peficitinib could not to be explained by blocking only secondary mediators. We therefore hypothesize that the effects of peficitinib and filgotinib at high concentrations are mediated by suppressing other kinases. Further, studies are required to examine the inhibition profile of JAKi in different human cell types at observed serum drug concentrations *in vivo*.

Our study indicates a possible advantage of peficitinib by targeting RASF *in vitro* and could result in higher response rates *in vivo* compared to other JAKi. Especially subgroups of patients suffering from severe synovial proliferations might benefit from higher dosages of peficitinib.

## Conclusions

In conclusion, all JAKi tested suppressed the inflammatory response induced by OSM and by transsignaling of IL-6 in RASF. Only peficitinib was able to modulate the IL-1β induced response of RASF and their proliferation *in vitro* at concentrations which are close to reported C_max_ values of well tolerated doses *in vivo*, but the underlying mechanism remains unclear. Furthermore, peficitinib highly suppressed the migration of RASF showing the potential of peficitinib to target RASF.

## Data Availability

The datasets generated for this study are available on request to the corresponding author.

## Author Contributions

MD, EN, KF, and UM-L designed the project. MD, IA, and M-LH were responsible for acquisition of data. MD, RH, KF, and EN analyzed and interpreted the data. SR collected the tissue samples and contributed to the project design. MD, RH, IA, EN, and UM-L contributed to drafting of the article. All authors contributed to manuscript revision, read, and approved the submitted version.

### Conflict of Interest Statement

The authors declare that the research was conducted in the absence of any commercial or financial relationships that could be construed as a potential conflict of interest.
